# Supplementary figures and images for: Population-Based Survival Analysis of Stage IVB Small-Cell Neuroendocrine Carcinoma in Comparison to Major Histological Subtypes of Cervical Cancer
Source: Curr Oncol. 2023 Oct 24;30(11):9428–36. doi: 10.3390/curroncol30110682 (PMC10670469; doi:10.3390/curroncol30110682)

Supplemental Figure S1

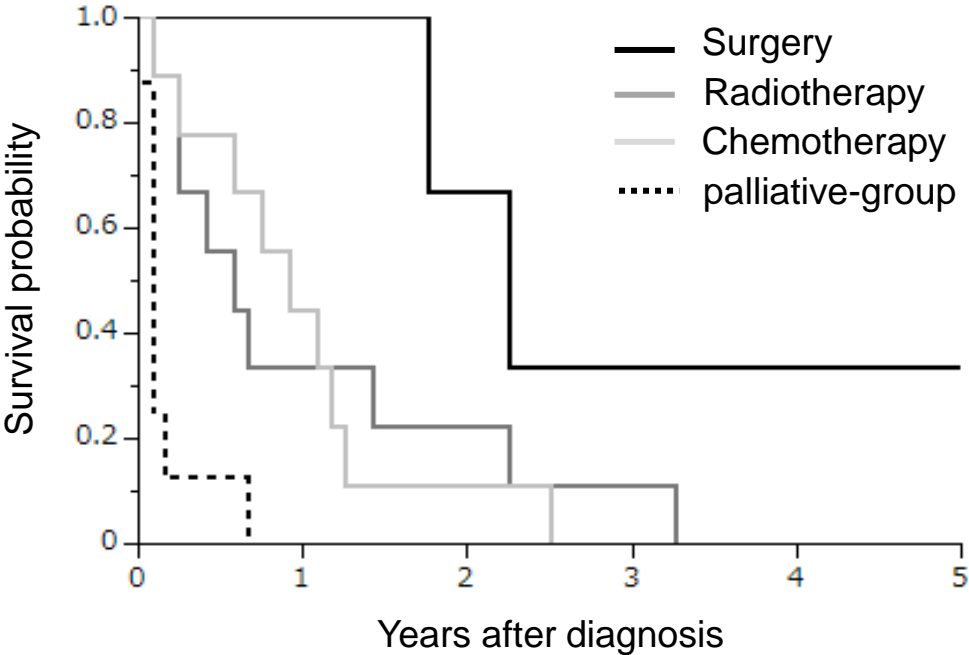

Supplement: Supplementary file 1 [file curroncol-30-00682-s001.zip › curroncol-2610684-supplementary.pdf]
